# Active Packaging Material Based on Immobilized Diatomaceous Earth/Zinc Oxide/High-Density Polyethylene Composite for Sea Food and Products

**DOI:** 10.3390/polym14235228

**Published:** 2022-12-01

**Authors:** Korakot Charoensri, Yang J. Shin, Kyu C. Kim, Hyun J. Park

**Affiliations:** 1 Department of Biotechnology, College of Life Sciences and Biotechnology, Korea University, 145 Anam-ro, Seongbuk-gu, Seoul 02841, Republic of Korea; 2 Negopack Co., Ltd., 197 Muha-ro, Pantan-myeon, Hwasung-si 18536, Republic of Korea

**Keywords:** active packaging, food packaging, seafood, antimicrobial, diatomaceous earth, zinc oxide

## Abstract

One of the key factors of supporting the rapidly expanding seafood product industry in terms of quality control is the utilization of active packaging materials. Microorganisms are primarily responsible for the perishability and rapid disintegration of seafood. The incorporation of an inorganic compound, such as silica-based diatomaceous earth (DE), and a metal oxide, such as zinc oxide (ZnO), is proposed to develop active packaging materials with excellent antibacterial activity, minimized fishy odor, and brittleness at subzero temperatures. The mechanical, morphological, and physicochemical properties of these materials were investigated. The results show that the addition of DE/ZnO improved the antibacterial activity of high-density polyethylene (HDPE) samples by up to approximately 95% against both gram-positive and -negative bacteria. Additionally, it enhanced the Izod strength and stability at subzero temperatures of the samples. The odor evaporation test revealed that trimethylamine can be minimized in proportion to increasing DE/ZnO composite concentration. As a result, the development of active packaging materials from DE/ZnO composites is an emerging polymeric packaging technology for seafood products, wherein packaging and seafood quality are linked.

## 1. Introduction

Aquaculture is the world’s most rapidly expanding food production industry. In 2021, the global fishery market is anticipated to increase in value by 12% and in volume by 3.7%, whereas production is expected to increase by 4 million tons between 2020 and 2022 [1,2,3]. In addition, worldwide aquaculture production must reach 109 million tons by 2030 and 140 million tons by 2050 to provide protein and essential nutrients for the global human diet [3,4,5]. To support the supply chain of seafood production, it is necessary to develop technologies to enhance and regulate the quality of seafood products [1,5,6]. Packaging science and technology is a crucial factor that must be continuously advanced. Seafood quality and fishy odors pose the greatest challenges for seafood packaging [1,7,8]. Seafood is classified as perishable owing to its high water content, which promotes rapid decomposition, particularly by microorganisms [9,10,11,12,13]. Improper handling during pre- and post-harvest hastens the growth of native microorganisms, which triggers chemical and biochemical reactions that result in deterioration [14,15,16,17]. Fishy odor impedes the packaging of seafood products as it is difficult to remove the fishy odor from water that permeates and adheres to the packaging of seafood products. Trimethylamine (TMA) is a volatile alkaline compound that is produced when bacteria reduce trimethylamine oxide to TMA [18,19]. Managing the fishy odors during transportation and storage is difficult and, to overcome this difficulty, we utilized the excellent properties of a composite of an inorganic compound, silica-based diatomaceous earth (DE), and zinc oxide (ZnO) with high-density polyethylene (HDPE) produced via injection molding. DE, an inexpensive adsorbent composed of 80–90% amorphous silica/ SiO_2_ is known to not be harmful to people and the environment [20,21,22]. It has a high absorption capacity and a low density because it contains many pores. In addition, the presence of a complex 3D porous structure makes it an excellent candidate for loading antimicrobial or antioxidant-active compounds [22,23]. According to previous reports, DE is utilized in a variety of applications, including the beverage industry for filtration, agricultural chemical additives, cosmetics, food, and plastics [24,25]. The Food and Drug Administration (FDA) classified DE as generally regarded as safe (GRAS) [25]. Similarly, ZnO is FDA-approved as GRAS [26]. ZnO is widely utilized in various fields because of its remarkable antimicrobial photocatalytic properties [26,27]. According to numerous reports, ZnO exhibits antimicrobial properties against both gram-positive and gram-negative bacteria. Several mechanisms have been proposed for the antibacterial activities of ZnO, including the release of metal ions, the interaction of ZnO nanoparticles with microorganisms, and the formation of reactive oxygen species (ROS) by the effect of light radiation [26,27,28]. The antimicrobial activity of ZnO is enhanced by increasing the surface area-to-volume ratio, which results in increased H_2_O_2_ production. Because of their high surface area, thermal stability, and eco-friendliness, microporous and mesoporous materials, such as silica and zeolite, have been used as host materials to produce ZnO particles that are small and have a distinctive structure [29,30,31,32]. 

In this study, we investigated a manufactured injection mold of HDPE/DE/ZnO with different relative compositions to determine its antibacterial activity, ability to minimize the fishy odor, and strength at subzero temperatures for the support and development of the seafood product packaging industry to meet the rising demand for seafood products in the global market.

## 2. Materials and Methods

### 2.1. Materials

Zinc chloride (ZnCl_2_) was purchased from Sam Chun Pure Chemicals (Gyeonggi, Republic of Korea). Commercial DE (Celite -CF-1031) was supplied by Tianjin Chemist Scientific, Ltd. (Tianjin, China). Sodium hydroxide (NaOH) was purchased from Daejung Chemicals Metals (Gyeonggi, Republic of Korea). Commercial HDPE resin had a melt flow rate of 4 g/ 10 min (190 °C, 2.16 kg) and a density of 0.955 g/cm^3^.

### 2.2. Elaboration of Antibacterial Material

DE (5 g) was suspended in deionized (DI) water (100 mL). A 2 and 4 wt% equivalent of ZnO derived from ZnCl_2_ was added to the suspension (the sample were referred to as DE/ZnO-I and DE/ZnO-II, respectively). For ion exchange, the slurry was stirred under continuous magnetic stirring at 80 °C for 5 h. Subsequently, 0.1 M NaOH was added dropwise until the pH reached 11. After 2 h, the product was filtered, washed several times with DI, centrifuged at 4000 rpm for 10 min, and dried overnight at 60 °C. The resulting product was then calcined for 2 h at 500 °C to obtain DE/ZnO powder. Prior to extrusion, DE/ZnO-II was selected according to the characterization performance and evaporated to dryness in a 90 °C hot-air oven. The DE/ZnO composite was prepared by mixing HDPE resin in the specified amounts (0, 2, and 4 wt%, and the samples were referred to as HDPE/ZnO, HDPE/ZnO_2_, and HDPE/ZnO_4_, respectively) with as-prepared DE/ZnO for 30 min using a planetary mixer. The mixture was extruded using a twin-screw extruder (LTE-20–40, Labtech Engineering) operated at 80–150 °C and 190 rpm. The HDPE/DE/ZnO extrudates were cut with a palletizer into 2.5 mm lengths (LZ-120, Lab tech Engineering). The resultant pellets were molded according to the requirements of each test specimen in an injection machine, Meteor 270/75 from Mateo & Sole (Barcelona, Spain) at 190 °C using a mirror-finishing steel mold with standard geometries for sample characterization. A 75-ton dampening force was applied, and the durations for cavity filling and cooling were set to 1 and 10 s, respectively.

### 2.3. Characterization of the DE/ZnO and HDPE/DE/ZnO Composite Material

#### 2.3.1. Characterization of the DE/ZnO Composite

The surface morphology and elemental composition of the pre-prepared DE/ZnO composite were determined using a combination of a transmission electron microscope (TEM, JEM-2100 HR, JEOL, Tokyo, Japan), field-emission scanning electron microscopy (FE-SEM, 508010, Hitachi, Japan), and energy dispersive spectroscopy (EDS) mapping. X-ray diffraction (XRD, JP/Max-3C, Rigaku, Japan) was used to examine the crystalline structures of the prepared DE/ZnO composite. To establish the Brunauer–Emmett–Teller (BET) surface area and pore size distribution, a QUADRASORB SI was used to archive the nitrogen sorption isotherm. The tensile strength (TS) and elongation at the break of the samples were evaluated using a universal testing machine (QMESYS, QT100T, Gyeonggi-do, Korea). Five samples were evaluated in accordance with the ASTM 0882–02 standard test procedure, and the average values were determined.

#### 2.3.2. Characterization of the HDPE/DE/ZnO Composite

The thermal characteristics of the materials were analyzed using thermogravimetric analysis (TGA, N-1000, SCINCO, Seoul, Republic of Korea). Specimens weighing between 15–20 mg were scanned between 100 and 600 °C at a heating rate of 10 °C/min in a nitrogen flow with a flow rate of 30 mL/min. The maximum degradation rate temperature was calculated at the peak of the first derivative (DTG) of the TGA curve. Izod impact and brittleness temperature tests were conducted to evaluate the mechanical qualities of the material to determine its potential packaging applications for seafood products. Izod tests were conducted using an Instron LEAST 9050 impact pendulum with a 0.5 J hammer in accordance with ASTM D256-10(2018). In addition, a temperature test for brittleness was conducted according to ASTM D746-20 (type I specimen) at −40° and −60 °C. Five specimens were tested for each sample and the results were average values. For the odor evaporation test, a 50 mm × 50 mm specimen was enclosed in a 5 L reactor. The initial concentration of the test gas was injected at 50 μmol/mol of TMA, and the concentration of the test gas was measured at 0, 30, 60, 90, and 120 min; this is referred to as the sample concentration. A gas-sampling pump (model GV-100S; Gastec Corp.) was used to collect and analyze samples of the gas emission, using detector tubes (SPS-KCLI2218-6218) for TMA. Throughout the experiment, the temperature was maintained at 23 °C, the relative humidity was 50% RH, and five replicate tests were carried out in the same conditions for each sample. The following formula was used to compute the concentration reduction rate for each time interval:

(1) $$\mathrm{Gas}\;\mathrm{concentration}\;(\%)=\frac{A-B}{A}\;\times \;100\;$$

where *A* is the concentration of the blank and *B* is the concentration of the sample.

### 2.4. Antimicrobial Study

The Antimicrobial activity of the synthesized DE/ZnO was evaluated using the agar disc diffusion method. All pathogenic strains (gram-negative strains; *Escherichia coli* (*E. coli*) and gram-positive strains; *Staphylococcus aureus* (*S. aureus*)) were cultured in Mueller–Hinton (MH) broth at 37 °C accompanied by shaking at 200 rpm until an optical density at 600 nm of 0.4–0.6 (exponential phase) was reached. The bacterial suspension was suspended in a saline solution, and 0.1 mL of each pathogenic strain was spread on MH agar. The DE/ZnO composite weighing 0.5 mg was suspended in 2 mL of 10% DMSO and dispersed on filter paper discs 6 mm in diameter. The loaded filter paper discs were placed on Petri dishes containing pathogenic strains and incubated at 37 °C. Antimicrobial activity was observed after 24 h by measuring the zone of inhibition, which was performed in triplicates. The broth microdilution method was used to determine the minimum inhibitory concentration (MIC). The MIC values were measured in LB medium using a 96-well plate. Serial dilutions were performed, and the final concentrations of the film solutions were 50, 25, 12.5, 6.25, 3.75, and 1.5 μg/mL. Samples in LB media without a microbial suspension were considered as a negative control. The 96-well plates were incubated at 37 °C for 24 h. The turbidity of the plate contents was interpreted as the growth of microorganisms and the appearance of the content in each well, in comparison with the appearance of the negative control samples. The lowest concentration that did not exhibit turbidity following incubation was interpreted as the MIC. To determine the minimum bactericidal concentration (MBC), the mixtures in each well that exhibited no turbidity were streaked onto MH agar and incubated at 37 °C for 24 h. The lowest concentration of the test substance that prevented colony formation was considered the MBC. A modified procedure compared with the ASTM (ASTM E 219-0) and JIS standard (JIS Z 2801:2020) was used to evaluate the antibacterial activity of the HDPE/DE/ZnO injection mold. Briefly, three pieces (50 mm × 50 mm) of each test specimen were placed on a Petri dish, inoculated with 400 μL of bacterial suspension (10^5^ CFU/mL; OD_600_ = 0.4), and coated with 40 mm × 40 mm pieces of uncoated sterile PP film. After incubation for 24 h at 37 °C, the bacteria were collected from the film surface using 10 mL of sterile saline which was serially diluted and planted on tryptic soy agar. Antimicrobial activity was expressed as a log reduction value which was calculated using the following equation:

Antimicrobial activity(log) = log(*A*) − log(*B*) (2)

where *A* is the number of viable microorganisms in the control sample and *B* is the number of viable microorganisms in the treatment sample.

### 2.5. Statical Analysis

Statistical analyses were performed using ANOVA with SPSS software (SPSS 25, IBM, Chicago, IL, USA). Duncan’s multiple range tests was used to indicate the statistical differences among the mean values. The results were considered statistically significant at *p* < 0.05. Data are presented as the mean ± standard deviation for each experiment.

## 3. Results and Discussion

### 3.1. Characterization of the DE/ZnO Composite

**Figure 1:**
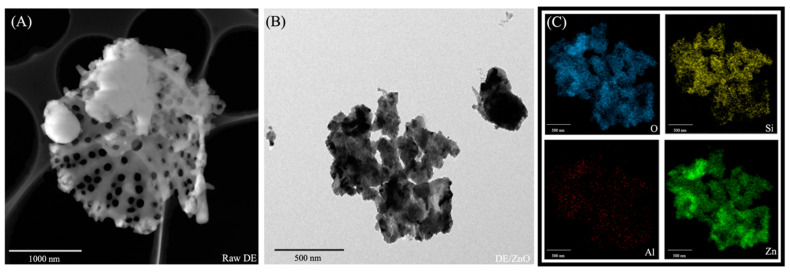
TEM image of raw DE (**A**), DE/ZnO-II (**B**), and EDS mapping image of DE/ZnO-II composite (**C**).

**Figure 2:**
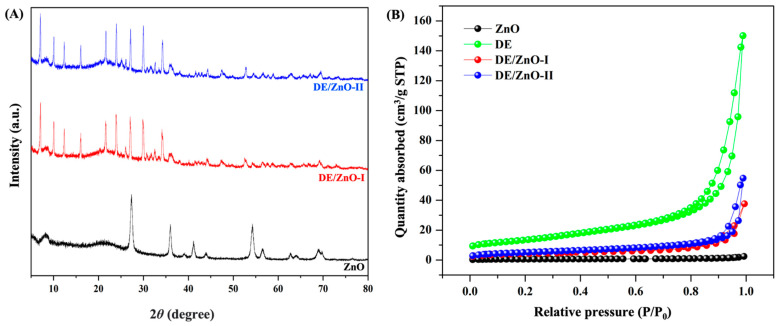
XRD pattern (**A**) and the BET isotherm (**B**) of ZnO and prepared DE/ZnO composite.

The TEM micrographs were utilized to evaluate the morphologies of the fabricated DE/ZnO composites. Raw DE (Figure 1A) has skeleton structures and pores ranging from 5 to 15 μm, similar to those of diatom algae; the diameters of the pores varied between 200 and 460 nm. Owing to the nanometric proportion and morphological structure inherent in DE, this material is a potential choice for antibacterial applications as a host for ZnO particles [33,34]. The TEM micrographs, as well as the EDS of the synthesized DE/ZnO composite, are shown in Figure 1B,C. Agglomerates of the ZnO composite were consistently distributed on the surface and within the porous architecture of the DE. In accordance with the EDS pattern, the presence of zinc confirmed that ZnO was evenly dispersed throughout the DE structure. The effect of homogenization on the crystalline structure of the DE/ZnO composite was revealed by XRD. The resulting XRD patterns are shown in Figure 2A. The characteristic peaks of the hexagonal wurtzite structure of ZnO (JCPDS:89-13971) are (100), (002), (101), and (102). The Wurtzite structure of ZnO is superimposed over the intense cristobalite silica peak (101) of frustules (JCPDS 76-0940), and the crystal development of the ZnO particles may be hinged on the DE support [35]. Figure 2B depicts the N_2_ adsorption–description isotherm of raw DE, ZnO, and DE/ZnO-I,II composites, while Table 1 summarizes the textural characteristics. DE/ZnO-I and DE/ZnO-II exhibited a standard type IV curve (IUPAC) classification; the small hysteresis loop at a high relative pressure level indicates the presence of a mesoporous structure. This is in agreement with the TEM observation which reveals the agglomerate structure of the DE/ZnO composite [28,29].

### 3.2. Characterization of the HDPE/DE/ZnO Injection Mold

**Figure 3:**
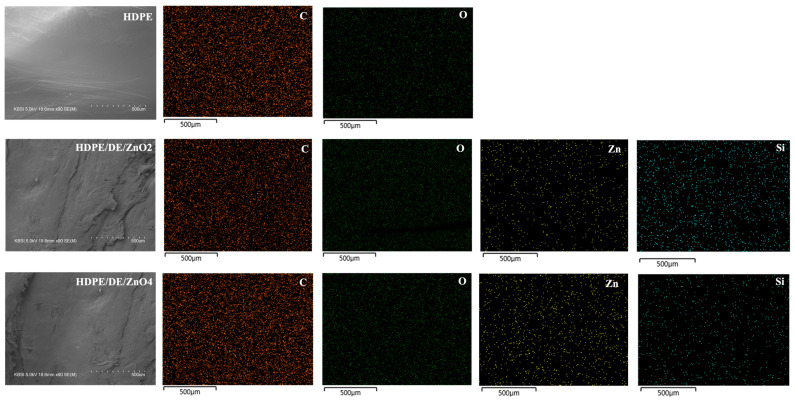
FE-SEM with an EDS mapping image of the neat HDPE and HDPE/DE/ZnO injection mold.

**Figure 4:**
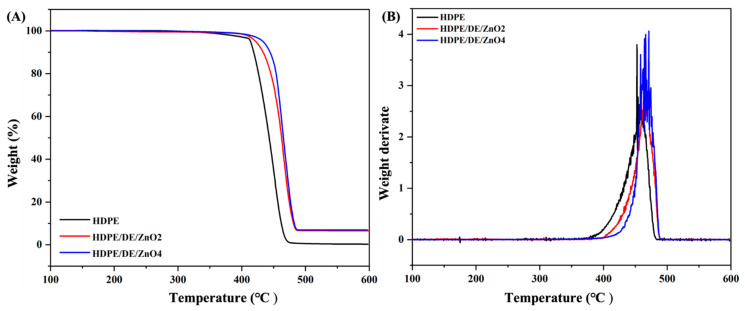
Thermogravimetric analysis (**A**) TGA and (**B**) DTG of the neat HDPE and HDPE/DE/ZnO injection mold.

Through EDS and SEM analyses, the existence of the DE/ZnO composite in the HDPE injection mold was confirmed. In Figure 3, it can be observed that the surface of the raw HDPE is smooth, in contrast to the surface of the injection mold containing the DE/ZnO composite, which has a rough surface. Additionally, one can observe aggregate elements all over the surface, which corresponds to the DE/ZnO composite, and the EDS results show that Zn and Si elements are well dispersed all over the surface of the test specimen [36,37]. Regarding thermal stability, Figure 4 shows the TGA and its first derivative (DTG) curves. Compared with the injection molds made from neat HDPE, the thermal stability of HDPE/DE/ZnO composites is greater, since neat HDPE decomposes substantially at 480 °C, losing over 90% of its mass, whereas 4% HDPE/DE/ZnO shows only approximately 80% mass loss in the same temperature range. Thermal analysis can be associated with the minimum volume fraction of particles incorporated into the composite, and the similar thermal properties of the matrix and composites suggest processing conditions analogous to those of HDPE in a hypothetical industrial-scale production scenario [38,39]. Table 2 displays the results of the Izod impact and stability test at freezing temperatures. Increasing the DE/ZnO composite content increased the impact strength of the composites. In addition, the impact strength at subzero temperatures remained nearly unchanged between −40 and −60 °C. Because HDPE is a ductile material, it is possible for the DE/ZnO composite to distribute the incoming stress equally and limit the probability of additional fatigue crack extension [40,41]. Table 2 summarizes the mechanical properties of HDPE and HDPE/DE/ZnO composites, including tensile strength, elongation at break, and elastic modulus. Due to the increase in intermolecular and intramolecular chain interactions of the HDPE matrix when mixed with the DE/ZnO nanocomposite, the HDPE mechanical characteristics did not statistically alter in elongation at break. Moreover, it could be assumed that the addition of DE/ZnO has no effect on the mechanical properties of HDPE [42,43,44].

### 3.3. Odor Test Minimization for the HDPE/DE/ZnO Injection Mold

**Figure 5:**
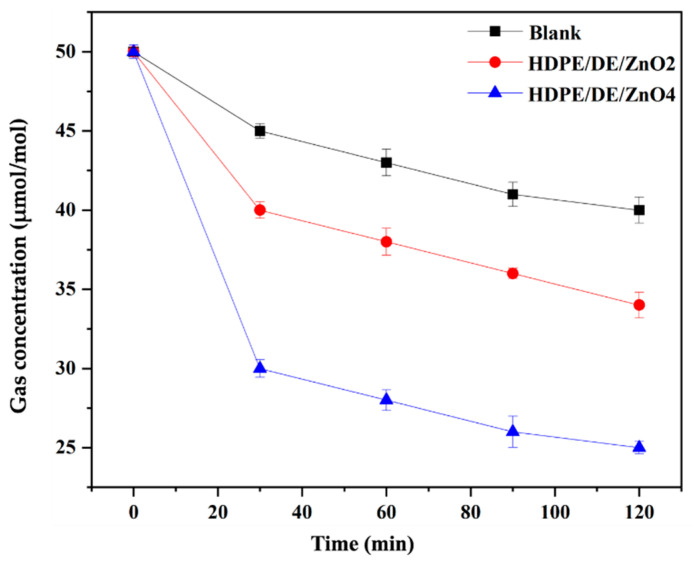
Trimethylamine deodorization test of the HDPE and HDPE/DE/ZnO injection mold.

The determination of the odor reduction test results is shown in Figure 5. From these results, it can be concluded that the DE/ZnO composite has a high effect on HDPE in reducing TMA up to 37.5% within 120 min, which is notably different from the test specimen of pure HDPE. In this study, TMA was considered a representative volatile chemical because it is primarily detected in seafood products, which meets the purpose of the study. DE and ZnO can be utilized as affordable odor sensors capable of detecting the freshness of foods and beverages because of their continual sensitivity to TMA. Due to its physicochemical property and porous structure, DE has efficient adsorbent properties, and it has been observed that it may effectively reduce the fishy odor during low-temperature adsorption. In addition, ZnO may dissociate hydrogen in a heterogeneous manner, and a ZnO-tailored matrix would increase the number of positively charged volatile molecules with high adsorbent material potential [45,46,47,48].

### 3.4. Antimicrobial Activity

The antibacterial activity of the synthesized DE/ZnO was investigated using the disc diffusion method with *E. coli* and *S. aureus* culture suspensions; the findings are shown in Table 3. Intriguingly, discs impregnated with DE/ZnO had a considerable surface area. This also indicates that increasing the DE/ZnO content has the potential to increase antimicrobial activity, which is considered to be a result of the combined effects of an increase in surface area, the release of Zn ions onto the bacterial cell wall, and electrostatic interaction between the surface and the microbial pathogens. To provide a more quantitative evaluation of antimicrobial activity, the MIC and MBC values were obtained (Table 3). The injection mold material with the lowest MIC and MBC values against *E.coli* and *S. aureus* samples was chosen based on the results. The antibacterial characteristics of the injection-molded HDPE/DE/ZnO composites were also explored. Neither *E. coli* nor *S. aureus* could be inhibited by the pure HDPE sample. HDPE/DE and HDPE/ZnO were also produced to assess the antibacterial efficacy of HDPE/DE/ZnO, but their antimicrobial potential is insufficient due to agglomeration and poor dispersion in the HDPE matrix. In contrast, the addition of DE/ZnO to the HDPE specimens significantly increased the antibacterial activity. Specifically, the inclusion of DE/ZnO at a 4 wt% increased the antibacterial response against *S. aureus* and *E. coli* by 1.5 and 1.3 log units, respectively. Based on these findings, we can describe the mechanism behind ZnO composite antibacterial action by referencing prior research [26,28,46]. The antibacterial action of metal particles is caused by multiple processes, including the production of reactive oxygen species, the release of cationic ions, and cell wall destruction. Numerous researchers have observed that, in the case of ZnO and Zn^2+^, ions can penetrate bacterial cell walls and interact with cytoplasmic material to kill bacteria [49,50]. We sought to clarify the interactions between the ZnO composite and bacteria in the interface. It is well known that the cell walls of bacteria have negative surface charges. According to our findings, the antibacterial activity of the DE/ZnO composite may result from both the formation of ROS and the consolidation or substitution of electrostatic contacts between ZnO particles and bacterial cell membranes [51,52,53,54].

## 4. Conclusions

The development of active packaging for seafood products is essential for the rapid expansion of the aquaculture industry. Active packaging materials based on DE/ZnO composites were developed in this study to improve their physical, chemical, and antibacterial properties. The characteristic results indicated that DE/ZnO was properly synthesized and disseminated in the HDPE sample. The addition of a DE/ZnO composite to HDPE improved its impact strength and stability at subzero temperatures between −40 °C and −60 °C. Additionally, it reduced the volatile chemical TMA, which is prevalent in seafood products. In addition, the antibacterial effectiveness of HDPE/DE/ZnO against both gram-negative (*E. coli*) and gram-positive *(S. aureus*) bacteria was exceptional. Based on the findings of this study, the use of this active packaging material for the production of packaging for seafood products is an easily accessible and inexpensive modification strategy.

## Figures and Tables

**Table 1:** The BET surface, pore volume, and pore size analysis.

Sample	BET Surface (m^2^/g)	Pore Volume (cm^3^/g)	Pore Size (nm)
DE	48.08 ± 2.13 ^a^	0.25 ± 0.06 ^a^	17.54 ± 1.61 ^b^
ZnO	2.28 ± 0.29 ^c^	0.01 ± 0.04 ^c^	12.17 ± 0.89 ^c^
DE/ZnO-I	15.88 ± 1.72 ^b^	0.05 ± 0.04 ^b^	18.42 ± 1.81 ^ab^
DE/ZnO-II	18.57 ± 1.41 ^b^	0.09 ± 0.05 ^b^	20.06 ± 2.11 ^a^

Note: values in the same column followed by different uppercase letters within a column are significantly different (*p* < 0.05) according to Duncan’s multiple range tests.

**Table 2:** Izod impact, stability at subzero temperature, trimethylamine deodorization, and mechanical property test value.

Sample	Izod Impact (J/m)	Stability at Subzero Temperature		Trimethylamine Deodorization Test at 120 Min		Tensile Strength (MPa)	Elongation (%)	Elastic Modulus (MPa)
		−40 °C	−60 °C	Gas Concentration (μmol/mol)	Reduction Rate (%)			
HDPE	51.00 ± 2.14 ^c^	×	×	40.21 ± 1.23 ^c^	11.32	25.94 ± 1.78 ^a^	54.25 ± 3.79 ^a^	0.48 ± 0.17 ^b^
HDPE/DE/ZnO_2_	104.00 ± 3.28 ^b^	O	O	34.13 ± 0.81 ^b^	15.43	26.29 ± 1.29 ^a^	50.85 ± 1.90 ^ab^	0.52 ± 0.48 ^a^
HDPE/DE/ZnO_4_	164.00 ± 5.12 ^a^	O	O	25.21 ± 0.37 ^a^	37.51	25.95 ± 1.22 ^a^	47.32 ± 1.28 ^b^	0.58 ± 0.21 ^a^

Note: values in the same column followed by different uppercase letters within a column are significantly different (*p* < 0.05) according to Duncan’s multiple range tests. (×)  indicates that the test specimen is altered during the test, whereas (O) indicates that it remains stable.

**Table 3:** Antimicrobial activity; the MIC and MBC activities against *Escherichia coli* (*E. coli*) and *Staphylococcus aureus* (*S. aureus*).

Sample	DE/ZnO	HDPE	HDPE/DE	HDPE/ZnO	HDPE/DE/ZnO_2_	HDPE/DE/ZnO_4_
Inhibition zone (mm)						
*E. coli*	10.12 ± 0.43 ^a^	-	-	-	-	-
*S. aureus*	9.83 ± 0.56 ^ab^	-	-	-	-	-
MIC (μg/mL)						
*E. coli*	25.00	-	-	-	-	-
*S. aureus*	12.50	-	-	-	-	-
MBC (μg/mL)						
*E. coli*	12.50	-	-	-	-	-
*S. aureus*	12.50	-	-	-	-	-
Antimicrobial activity (%)						
*E. coli*	99.99	-	70.13	84.73	93.69	97.08
*S. aureus*	99.99	-	67.18	80.12	74.21	95.32

Note: values in the same column followed by different uppercase letters within a column are significantly different (*p* < 0.05) according to Duncan’s multiple range tests.

## Data Availability

Not applicable.

## References

[1] Laorenza Y., Chonhenchob V., Bumbudsanpharoke N., Jittanit W., Sae-Tan S., Rachtanapun C., Chanput W.P., Charoensiddhi S., Srisa A., Promhuad K. (2022). Polymeric Packaging Applications for Seafood Products: Packaging-Deterioration Relevance, Technology and Trends. Polymers.

[2] Ahmed N., Azra M.N. (2022). Aquaculture Production and Value Chains in the COVID-19 Pandemic. Curr. Environ. Health Rep..

[3] Gosh K., Chowdhury S., Acharjee D.C., Mamun A.-A., Ghosh R. (2022). Assessing the Economic Impacts of COVID-19 on the Aquaculture and Fisheries Sectors in Relation to Food Security: A Critical Review. Sustainability.

[4] Pita P., Ainsworth G.B., Alba B., Anderson A.B., Antelo M., Alós J., Artetxe I., Baudrier J., Castro J.J., Chicharro B. (2021). First Assessment of the Impacts of the COVID-19 Pandemic on Global Marine Recreational Fisheries. Front. Mar. Sci..

[5] Kontominas M., Badeka A., Kosma I., Nathanailides C. (2021). Recent Developments in Seafood Packaging Technologies. Foods.

[6] Gokoglu N. (2020). Innovations in Seafood Packaging Technologies: A Review. Food Rev. Int..

[7] Ye B., Chen J., Fu L., Wang Y. (2022). Application of nondestructive evaluation (NDE) technologies throughout cold chain logistics of seafood: Classification, innovations and research trends. LWT.

[8] Olatunde O.O., Shiekh K.A., Benjakul S. (2021). Pros and cons of cold plasma technology as an alternative non-thermal processing technology in seafood industry. Trends Food Sci. Technol..

[9] Tavares J., Martins A., Fidalgo L., Lima V., Amaral R., Pinto C., Silva A., Saraiva J. (2021). Fresh Fish Degradation and Advances in Preservation Using Physical Emerging Technologies. Foods.

[10] Abd El-Hay M.M., Jafari S.M. (2022). 10—Processing and preparation of fish. Postharvest and Postmortem Processing of Raw Food Materials.

[11] Getu A., Misganaw K. (2015). Post-harvesting and Major Related Problems of Fish Production. Fish. Aquac. J..

[12] Fatima A., Yasir S., Khan M.S., Manan S., Ullah M.W., Ul-Islam M. (2021). Plant extract-loaded bacterial cellulose composite membrane for potential biomedical applications. J. Bioresour. Bioprod..

[13] Madni A., Kousar R., Naeem N., Wahid F. (2021). Recent advancements in applications of chitosan-based biomaterials for skin tissue engineering. J. Bioresour. Bioprod..

[14] Gu J.-D., Katayama Y., Joseph E. (2021). Microbiota and Biochemical Processes Involved in Biodeterioration of Cultural Heritage and Protection. Microorganisms in the Deterioration and Preservation of Cultural Heritage.

[15] Singh S., Shin Y., Lee Y.S. (2016). Antimicrobial seafood packaging: A review. J. Food Sci. Technol. Mysore.

[16] Sheng L., Wang L. (2021). The microbial safety of fish and fish products: Recent advances in understanding its significance, contamination sources, and control strategies. Compr. Rev. Food Sci. Food Saf..

[17] Thomas A., Konteles S.J., Ouzounis S., Papatheodorou S., Tsakni A., Houhoula D., Tsironi T. (2021). Bacterial community in response to packaging conditions in farmed gilthead seabream. Aquac. Fish..

[18] Ishida T. (2020). Removal of Fish Odors Form Styrofoam Packaging to Improve Recycling Potential Using Hansen Solubility Parameters. Recycling.

[19] Schmidt A.C., Hebels E., Weitzel C., Kletzmayr A., Bao Y., Steuer C., Leroux J. (2020). Engineered Polymersomes for the Treatment of Fish Odor Syndrome: A First Randomized Double Blind Olfactory Study. Adv. Sci..

[20] Grommersch B.M., Pant J., Hopkins S.P., Goudie M.J., Handa H. (2018). Biotemplated Synthesis and Characterization of Mesoporous Nitric Oxide-Releasing Diatomaceous Earth Silica Particles. ACS Appl. Mater. Interfaces.

[21] Ruíz-Baltazar Á.d.J. (2018). Green Composite Based on Silver Nanoparticles Supported on Diatomaceous Earth: Kinetic Adsorption Models and Antibacterial Effect. J. Clust. Sci..

[22] Bellotti N., Deyá C., Atta ur R. (2019). Chapter 14—Natural Products Applied to Antimicrobial Coatings. Studies in Natural Products Chemistry.

[23] Fernández M., Bellotti N. (2017). Silica-based bioactive solids obtained from modified diatomaceous earth to be used as antimicrobial filler material. Mater. Lett..

[24] Zhang Y., Duan C., Bokka S.K., He Z., Ni Y. (2022). Molded fiber and pulp products as green and sustainable alternatives to plastics: A mini review. J. Bioresour. Bioprod..

[25] Cacciotti I., Mori S., Cherubini V., Nanni F. (2018). Eco-sustainable systems based on poly(lactic acid), diatomite and coffee grounds extract for food packaging. Int. J. Biol. Macromol..

[26] Charoensri K., Rodwihok C., Wongratanaphisan D., Ko J., Chung J., Park H. (2021). Investigation of Functionalized Surface Charges of Thermoplastic Starch/Zinc Oxide Nanocomposite Films Using Polyaniline: The Potential of Improved Antibacterial Properties. Polymers.

[27] Charoensri K., Rodwihok C., Ko S.H., Wongratanaphisan D., Park H.J. (2021). Enhanced antimicrobial and physical properties of poly (butylene adipate-co-terephthalate)/zinc oxide/reduced graphene oxide ternary nanocomposite films. Mater. Today Commun..

[28] Rodwihok C., Suwannakeaw M., Charoensri K., Wongratanaphisan D., Woo S.W., Kim H.S. (2021). Alkali/zinc-activated fly ash nanocomposites for dye removal and antibacterial applications. Bioresour. Technol..

[29] Azizi-Lalabadi M., Ehsani A., Divband B., Alizadeh-Sani M. (2019). Antimicrobial activity of Titanium dioxide and Zinc oxide nanoparticles supported in 4A zeolite and evaluation the morphological characteristic. Sci. Rep..

[30] Li M., Wu L., Zhang Z., Mai K. (2017). Preparation of ZnO-supported 13X zeolite particles and their antimicrobial mechanism. J. Mater. Res..

[31] Al-Tayyar N.A., Youssef A.M., Al-Hindi R.R. (2020). Antimicrobial packaging efficiency of ZnO-SiO2 nanocomposites infused into PVA/CS film for enhancing the shelf life of food products. Food Packag. Shelf Life.

[32] Hanula M., Pogorzelska-Nowicka E., Pogorzelski G., Szpicer A., Wojtasik-Kalinowska I., Wierzbicka A., Półtorak A. (2021). Active Packaging of Button Mushrooms with Zeolite and Açai Extract as an Innovative Method of Extending Its Shelf Life. Agriculture.

[33] Yang B., Liu X., Ma Z., Wang Q., Yang J. (2021). Synthesis of Nano-ZnO/Diatomite Composite and Research on Photoelectric Application. Catalysts.

[34] Dai X., Zeng H., Jin C., Rao J., Liu X., Li K., Zhang Y., Yu Y., Zhang Y. (2021). 2D–3D graphene-coated diatomite as a support toward growing ZnO for advanced photocatalytic degradation of methylene blue. RSC Adv..

[35] Rahmat F.I., Fen Y.W., Anuar M.F., Omar N.A.S., Zaid M.H.M., Matori K.A., Khaidir R.E.M. (2021). Synthesis and Characterization of ZnO-SiO2 Composite Using Oil Palm Empty Fruit Bunch as a Potential Silica Source. Molecules.

[36] Aggrey P., Nartey M., Kan Y., Cvjetinovic J., Andrews A., Salimon A.I., Dragnevski K.I., Korsunsky A.M. (2021). On the diatomite-based nanostructure-preserving material synthesis for energy applications. RSC Adv..

[37] Darvish M., Ajji A. (2021). Effect of Polyethylene Film Thickness on the Antimicrobial Activity of Embedded Zinc Oxide Nanoparticles. ACS Omega.

[38] Hidalgo-Salazar M.A., Correa-Aguirre J.P., García-Navarro S., Roca-Blay L. (2020). Injection Molding of Coir Coconut Fiber Reinforced Polyolefin Blends: Mechanical, Viscoelastic, Thermal Behavior and Three-Dimensional Microscopy Study. Polymers.

[39] Fiedot-Toboła M., Dmochowska A., Jędrzejewski R., Stawiński W., Kryszak B., Cybińska J. (2021). Pectin-organophilized ZnO nanoparticles as sustainable fillers for high-density polyethylene composites. Int. J. Biol. Macromol..

[40] LeBlanc J., Cavallaro P., Torres J., Ponte D., Warner E., Saenger R., Mforsoh I.N., Shukla A. (2020). Low temperature effects on the mechanical, fracture, and dynamic behavior of carbon and E-glass epoxy laminates. Int. J. Lightweight Mater. Manuf..

[41] Okayasu M., Tsuchiya Y. (2019). Mechanical and fatigue properties of long carbon fiber reinforced plastics at low temperature. J. Sci. Adv. Mater. Devices.

[42] Xu T.-C., Wang C.-S., Hu Z.-Y., Zheng J.-J., Jiang S.-H., He S.-J., Hou H.-Q. (2022). High Strength and Stable Proton Exchange Membrane Based on Perfluorosulfonic Acid/Polybenzimidazole. Chin. J. Polym. Sci..

[43] Fang D., Yan B., Agarwal S., Xu W., Zhang Q., He S., Hou H. (2021). Electrospun Poly[poly(2,5-benzophenone)]bibenzopyrrolone/polyimide nanofiber membrane for high-temperature and strong-alkali supercapacitor. J. Mater. Sci..

[44] Alsayed Z., Awad R., Badawi M.S. (2020). Thermo-mechanical properties of high density polyethylene with zinc oxide as a filler. Iran. Polym. J..

[45] Ayoub I., Kumar V., Abolhassani R., Sehgal R., Sharma V., Sehgal R., Swart H.C., Mishra Y.K. (2022). Advances in ZnO: Manipulation of defects for enhancing their technological potentials. Nanotechnol. Rev..

[46] Siddiqi K.S., Rahman A.U., Tajuddin, Husen A. (2018). Properties of Zinc Oxide Nanoparticles and Their Activity Against Microbes. Nanoscale Res. Lett..

[47] Lee C.-S., Kim I.-D., Lee J.-H. (2013). Selective and sensitive detection of trimethylamine using ZnO–In_2_O_3_ composite nanofibers. Sensors Actuators B Chem..

[48] Chung K.-H., Lee K.-Y. (2009). Removal of trimethylamine by adsorption over zeolite catalysts and deodorization of fish oil. J. Hazard. Mater..

[49] Mao H., Zhang B., Nie Y., Tang X., Yang S., Zhou S. (2021). Enhanced antibacterial activity of V-doped ZnO@SiO_2_ composites. Appl. Surf. Sci..

[50] Xia Y., Jiang X., Zhang J., Lin M., Tang X., Zhang J., Liu H. (2017). Synthesis and characterization of antimicrobial nanosilver/diatomite nanocomposites and its water treatment application. Appl. Surf. Sci..

[51] Tiwari V., Mishra N., Gadani K., Solanki P., Shah N.A., Tiwari M. (2018). Mechanism of Anti-bacterial Activity of Zinc Oxide Nanoparticle Against Carbapenem-Resistant Acinetobacter baumannii. Front. Microbiol..

[52] Mendes C.R., Dilarri G., Forsan C.F., Sapata V.d.M.R., Lopes P.R.M., de Moraes P.B., Montagnolli R.N., Ferreira H., Bidoia E.D. (2022). Antibacterial action and target mechanisms of zinc oxide nanoparticles against bacterial pathogens. Sci. Rep..

[53] Sirelkhatim A., Mahmud S., Seeni A., Kaus N.H.M., Ann L.C., Bakhori S.K.M., Hasan H., Mohamad D. (2015). Review on Zinc Oxide Nanoparticles: Antibacterial Activity and Toxicity Mechanism. Nano Micro Lett..

[54] Mocanu A., Isopencu G., Busuioc C., Popa O.-M., Dietrich P., Socaciu-Siebert L. (2019). Bacterial cellulose films with ZnO nanoparticles and propolis extracts: Synergistic antimicrobial effect. Sci. Rep..

